# Volontaire sain et recherche clinique au Sud : une approche communautaire

**DOI:** 10.48327/mtsi.v6i1.2026.823

**Published:** 2026-02-23

**Authors:** Jean-Philippe CHIPPAUX

**Affiliations:** IRD – MERIT, Université Paris Cité, F-75006 Paris, France

**Keywords:** Essai clinique, Recherche clinique, Éthique, Morale, Autonomie, Justice, Équité, Bénéfice, Risque, Occidental, Non-occidental, Volontaire sain, Patient, Clinical trial, Clinical research, Ethics, Morality, Autonomy, Justice, Equity, Benefit, Risk, Western, Non-Western, Healthy volunteer, Patient

## Abstract

**Contexte et objectifs:**

La participation des volontaires sains est indispensable dans de nombreuses études cliniques. Cependant, elle ne fait pas l’objet de recommandations éthiques spécifiques, alors que leur situation est différente de celle des patients. Contrairement à ces derniers, les volontaires sains ne sont pas en quête d’une intervention. En s’exposant aux aléas d’un essai clinique, ils font face à une balance bénéfices/risques défavorable. La charte VoIREthics publiée par l’Inserm et ses partenaires rappelle les principes de protection dont doivent bénéficier les volontaires sains participant à la recherche clinique. Essentiellement fondés sur la morale occidentale, ces principes peuvent entrer en conflit avec les morales autres qu’occidentales. La réflexion présentée ici souligne les difficultés qui pourraient survenir au cours de recherches cliniques en fonction du contexte culturel, législatif et réglementaire.

**Méthode:**

Les recommandations de la charte VolREthics ont été examinées, en fonction des principes d’autonomie, de bienfaisance et de justice, à la lumière de la littérature et d’expériences personnelles pour illustrer les différences de perception pouvant exister entre les sociétés occidentales et non-occidentales.

**Résultats et discussion:**

La place de la personne dans la société peut constituer un obstacle à son libre-arbitre. Dans certaines sociétés, l’autonomie individualiste est remplacée par une dépendance à la communauté. La perception de l’équilibre bénéfice/risque doit être rapportée à l’échelle de cette dernière et non à celle de l’individu. Dans la médecine traditionnelle, l’efficacité thérapeutique ne résulte pas du seul traitement et le risque thérapeutique n’est pas compris comme dans la médecine occidentale. En outre, les volontaires sains doivent représenter la diversité de la population. Si leur sélection résulte d’un consensus avec la communauté, les critères d’inclusion ne peuvent échapper au contrôle de l’investigateur ou du promoteur.

**Conclusion:**

Les principes rappelés dans la charte VolREthics doivent être contextualisés pour qu’ils soient acceptés par les parties prenantes de l’étude et pour éviter tout conflit avec les pratiques locales. Cependant, plutôt que l’application systématique de recommandations difficiles à adapter à tous les contextes, il est essentiel de discuter, point par point, de leur pertinence locale et de s’assurer du bien-être des participants à une étude clinique et de leur adhésion au protocole de recherche.

## Abstract (en)

**Background and objectives:**

The participation of healthy volunteers is essential for many clinical studies. However, their situation differs from that of patients, yet they are not subject to specific ethical recommendations. Unlike patients, healthy volunteers are not seeking treatment. They are exposed to the uncertainties of a clinical trial and face with an unfavorable risk/ benefit ratio. The VoIREthics charter, published by Inserm and its partners, outlines the protection principles that should protect healthy volunteers participating in clinical research. However, these principles are essentially based on Western morality and may therefore conflict with non-Western moral codes. The discussion presented here highlights the difficulties that may arise during clinical research depending on the cultural, legislative and regulatory context.

**Method:**

The VolREthics charter’s recommendations were examined based on the principles of autonomy, beneficence and justice. This examination was conducted in light of literature and personal experience, to illustrate the differences in perception that may exist between Western and non-Western societies.

**Results and discussion:**

A person’s place in society can limit their free will. In some societies, individual autonomy is replaced by dependence on the community. The perception of the benefit/risk balance must relate to the scale of the community, not the individual. In traditional medicine, therapeutic efficacy does not result from treatment alone, and therapeutic risk is not understood in the same way as in Western medicine. Furthermore, healthy volunteers must reflect the diversity of the population. If they are selected through a community consensus, the inclusion criteria cannot escape the investigator’s or sponsor’s control.

**Conclusion:**

The principles set out in the VolREthics charter must be contextualized to ensure acceptance by the study stakeholders and to avoid conflict with local practices. Rather than applying recommendations that are difficult to adapt to all contexts systematically, it is essential to discuss their local relevance point by point to ensure the well-being of clinical trial participants and their adherence to the research protocol.

**Figure:**
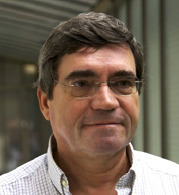

## Introduction

La participation d’un sujet sain – aux plans physique et mental – est indispensable dans de nombreux domaines de la recherche en santé, notamment lors des essais cliniques. Le volontaire sain se définit comme un participant à la recherche clinique ne sollicitant pas de recours à un traitement ou de prise en charge médicale, quels que soient son genre et son âge, même si cela influe sur les risques et les particularités de l’investigation. De même, l’objectif de la recherche clinique et la pathologie concernée constituent des enjeux spécifiques et ne sont pas considérés dans cet article.

Inclus lors du développement clinique d’un produit de santé^1^, les volontaires sains contribuent au progrès scientifique. Ils permettent de collecter des informations de référence sans qu’une pathologie n’interfère avec le produit ou ne biaise les résultats. Lors de la mise au point d’une intervention préventive ou thérapeutique, ils en mesurent l’innocuité, les effets indésirables et la pharma-cocinétique avant l’administration à des malades. D’autres caractéristiques ou propriétés, comme l’acceptabilité, la posologie, l’applicabilité, les associations thérapeutiques, feront l’objet de phases ultérieures et concerneront des sujets non-sains, sauf rares exceptions qui n’altèrent pas la suite de cet article. Dans certains cas (essais vaccinaux, notamment), les volontaires sains fournissent les premiers arguments d’efficacité. Sur la base des résultats préliminaires, la poursuite des essais cliniques pourra être autorisée.

Malgré l’importance du volontaire sain dans les études de prévention et de prophylaxie ainsi que dans les essais thérapeutiques de phase I, les modalités spécifiques de leur participation sont rarement précisées. Dans la plupart des recommandations nationales et internationales, les volontaires sains ne sont pas dissociés des patients inclus dans les études cliniques [25,27]. Pourtant, ils s’en distinguent par l’absence d’avantage médical direct et la menace d’une atteinte à leur bienêtre, voire à leur état de santé, conduisant à une balance bénéfices-risques déséquilibrée. En outre, la perspective d’une compensation financière les expose à être exploités [8,16,19].

La charte VoIREthics^2^ proposée par l’Inserm et ses partenaires internationaux précise les conditions d’inclusion des volontaires sains dans une étude clinique, notamment leur consentement libre à la suite d’une information détaillée sur les objectifs, le protocole et les résultats attendus de l’étude. Elle comporte 15 articles qui complètent les dispositions existantes en matière d’éthique et de protection des volontaires sains recrutés dans des études ou essais cliniques. Elle décrit les voies et moyens permettant de les protéger, de prévenir toute forme d’exploitation, de préjudices physiques ou moraux et de restriction de leur bien-être. La charte utilise une définition restreinte du terme « volontaire sain ». Elle se concentre spécifiquement sur les volontaires sains qui participent aux recherches interventionnelles portant sur un produit de santé sans bénéfice direct potentiel pour les participants. Cette approche est justifiée par le fait que ce type d’essai peut exposer les volontaires sains aux risques les plus élevés de préjudice, d’exploitation et de mise en danger de leur santé. Néanmoins, dans cet article, j’étendrai la définition du volontaire sain à tout sujet participant à une recherche clinique interventionnelle avec ou sans bénéfice individuel – y compris les personnes participant à des essais vaccinaux dont elles peuvent retirer un certain bénéfice alors qu’elles sont exclues de la définition de la charte –, dans la mesure où cette notion n’est pas comprise de façon identique selon les cultures. La participation d’un volontaire sain à la recherche clinique questionne 4 critères couverts par les principes d’autonomie, de bienfaisance et de justice : son indépendance par rapport aux acteurs de la recherche clinique, le bénéfice qu’il en tire, l’évaluation des risques et dommages qu’il faudra éviter ou indemniser et l’information préalable au consentement.

Cependant, la charte VoIREthics se fonde sur une approche universaliste et individualiste de l’éthique pour qui l’individu prime sur l’environnement et la collectivité. Dans de nombreux pays du Sud, il existe d’autres morales tout aussi légitimes, parfois également très anciennes, même si elles ne sont pas toujours entérinées par des textes formalisés et appartiennent parfois à la tradition orale. Elles apportent un éclairage particulier à certains paradigmes de l’éthique occidentale, notamment l’autonomie de la personne et le bénéfice individuel. Ces concepts concernent autant les patients que les volontaires sains, mais ces derniers présentent des particularités trop souvent négligées dans les lois et règlementations de nombreux pays, la France étant une exception notable depuis la loi Huriet-Sérusclat de 1988.

Cet article ne cherche pas à opposer, expliquer, commenter ou justifier le relativisme éthique. Elle vise à souligner la perception du volontaire sain qui peut être différente en raison d’une morale et d’une culture différentes de celles du promoteur ou des investigateurs.

## Approches méthodologiques

Ces commentaires sont issus d’une présentation faite devant le comité d’éthique de l’Inserm lors d’une discussion sur la charte VolREthics^3^. Ils se fondent sur une expérience personnelle acquise au cours d’essais cliniques menés en Afrique subsaharienne (Bénin, Cameroun, Guinée, Niger, Sénégal) et en Amérique du Sud (Bolivie, Brésil) depuis la fin des années 1980. Il n’y a pas eu de recherche bibliographique systématique et ciblée mais une exploitation de références sur les aspects philosophiques et éthiques de la recherche clinique.

Mes commentaires concernent tous les sujets participant à la recherche clinique, soulignant les spécificités propres aux volontaires sains, en me référant aux influences de certaines morales non-occidentales, notamment d’Afrique subsaharienne.

## Autonomie du volontaire sain

La décision de participer ou non à une étude clinique pourvu qu’il en ait la compétence légale, la capacité intellectuelle et physique et qu’il reçoive une information appropriée, renvoie au libre-arbitre du sujet.

Il serait illusoire de penser que l’information préalable suffit à garantir un choix libre et congruent. Des motivations complexes influencent la décision. Certaines sont circonstancielles, comme l’éventualité d’une rétribution – sujet traité avec pertinence dans la charte VoIREthics –, d’autres métaphysiques comme l’appartenance à une communauté [7]. Ces dernières ne sont pas abordées par la charte VoIREthics.

Dans de nombreuses sociétés du Sud – mais aussi quelques collectivités de pays industrialisés –, l’autonomie de la personne se construit par rapport à sa position et son statut dans la communauté, notamment du groupe socioécono-mique auquel il appartient [34]. L’individu, qu’il en ait conscience ou pas, n’est pas dissociable de la communauté dont il dépend [30]. Celle-ci, en fonction de ses intérêts ou convictions, dicte sa décision qui limite la liberté et la détermination individuelles, au nom de l’ordre social [11,31]. Le consentement exigé lors de l’inclusion dans une étude clinique se décline par rapport à la communauté à laquelle il appartient, parfois de sa place dans l’écosystème, mais non dans l’absolu [27]. Il ne peut donc y avoir une totale indépendance du sujet face au groupe dont il est membre, en opposition à la liberté individuelle défendue par les idéologies modernes [26]. Cette perspective modifie la perception et l’application de l’éthique telle qu’elle est conçue dans la médecine occidentale : la décision individuelle est subordonnée au bien commun. Elle questionne, en tout cas, l’universalité de l’éthique et de son application intangible quels que soient la personne, le lieu et le temps.

Cependant, cette situation n’est pas figée. De multiples sources d’information et de processus d’individualisation se développent face à la réalité quotidienne, aux tensions économiques, sociales, politiques et à la mondialisation qui désorganisent la communauté et distendent les liens entre ses membres, particulièrement dans le contexte d’une urbanisation croissante [4]. Le développement des réseaux sociaux s’étend à l’ensemble des communautés du Sud, de façon peut-être plus prégnante en milieu urbain que rural. La circulation de rumeurs, la pression des mouvements complotistes – avec ses spécificités culturelles et des ressentiments se référant à l’histoire ancienne ou récente – peuvent fortement influencer la décision du volontaire sain, y compris après son inclusion. De plus, la désinformation entraîne la perte de confiance envers les autorités politiques ou administratives et les institutions [20]. La controverse sur la vaccination (antivax) qui s’est développée en Afrique subsaharienne depuis les débuts du programme élargi de vaccination et s’est renforcée lors de la pandémie de Covid-19, en est un exemple manifeste. Ici aussi, une attention toute particulière doit être portée pour prendre en compte la vulnérabilité des personnes dans ce qui peut être assimilé à une crise identitaire. Le principe d’autonomie s’étend aux patients comme aux volontaires sains inclus dans les essais cliniques. Cependant, la motivation du volontaire sain n’est pas médicale. Elle est autre et cela doit être intégré dans l’information qui lui est délivrée comme dans le déroulement de l’étude clinique. En l’absence de bénéfice direct, le volontaire sain peut attendre un bénéfice collectif en faveur de la communauté à laquelle il appartient, à condition qu’il soit préalablement défini et, si les essais cliniques valident le nouveau produit, organisé (Encadré). Enfin, on ne peut exclure que le volontaire sain ne soit attiré par des avantages matériels ou financiers [3].

## Bénéfice du volontaire sain et évaluation du risque

Le bénéfice tiré de l’expérimentation à laquelle participe un sujet se réfère au principe de bienfaisance. À cet égard, la charte VoIREthics souligne dans son article 8 la notion de bien-être des volontaires sains. Au-delà de la nécessité de veiller à l’absence de préjudice physique et de risque d’exploitation, cette notion importante est relativement originale. Concernant le respect des personnes, parfois en situation de vulnérabilité, elle met en avant l’attention qui doit être portée à leur bien-être physique et mental.

Certains lui substituent le principe de non-malfaisance (d’abord, ne pas nuire) qui est tout aussi difficile à interpréter et à appliquer. Entre l’engagement actif (bienfaisance) et le revers passif (non-malfaisance), on peut voir non une complémentarité mais une dérobade [1]. L’équilibre bienfaisance/non-malfaisance renvoie à l’évaluation du rapport bénéfice/risque (efficacité/effets indésirables).

Encadré : Essai clinique du vaccin contre l’hépatite B à Niakhar, Sénégal

Dès les années 1960, l’observatoire de population de Niakhar situé dans le bassin arachidier du Sénégal a hébergé des essais cliniques [6,9,28]. À la fin des années 1970, un essai clinique d’un nouveau vaccin contre l’hépatite B constitué d’un concentré de particules AgHBs, y a été réalisé pour éliminer le cancer primitif du foie dont il est l’une des étiologies majeures [22]. Les essais cliniques de ce vaccin (Hevac B Pasteur) mis au point par Maupas et développé par Institut Pasteur Production, ont été conduits par l’université de Tours et celle de Dakar [22,23]. Bien que prévue lors de la mise en place de l’essai clinique, l’intégration de l’Hevac B Pasteur aux vaccins du programme élargi de vaccination n’a pas été réalisée pour des raison logistiques et financières, ce qui a entraîné une formidable déception affectant lourdement la poursuite des programmes de l’observatoire [23]. Un scénario similaire a été observé lors du développement d’un vaccin conjugué méningococcique A-C à Niamey à la fin des années 1990. Là encore, les habitants de Niamey n’ont pas eu accès au vaccin après la démonstration de son efficacité et de sa bonne tolérance chez les nouveau-nés nigériens.

La mise à disposition d’un traitement validé par les essais cliniques se présente différemment dans les sociétés favorisant l’accès aux soins et aux médicaments selon des mécanismes appropriés (sécurité sociale, mutuelles, assurances, etc.).

La Déclaration d’Helsinki accorde une légitimité à l’expérimentation si le rapport bénéfice/risque est favorable au sujet^4^. Équilibré, voire nettement bénéfique chez le patient qui espère une amélioration de sa santé ou, parfois, une guérison, il devient objectivement défavorable pour le volontaire sain qui n’attend pas de bénéfice direct mais dont la santé peut être altérée par l’intervention^5^, ce que ne souligne d’ailleurs pas suffisamment la dernière version de la déclaration d’Helsinki [3]. Si l’on y ajoute les risques et dommages éventuels entraînés par la participation d’un volontaire sain à un essai clinique, le principe de non-malfaisance semble plus approprié. À cet égard, les risques et contraintes des essais cliniques ne sont pas maîtrisés par les compagnies d’assurance en Afrique subsaharienne, pas plus que les participants ne connaissent leurs droits en cas de dommage, ce qui peut entraîner des difficultés en matière d’indemnisation ou de réparation. Il n’en reste pas moins que l’application du principe de bienfaisance aux volontaires sains peut satisfaire la communauté si celle-ci espère tirer un bénéfice collectif de la participation de quelques-uns de ses membres à la recherche clinique (Encadré). Fort de son indépendance par rapport au promoteur et à l’investigateur de l’étude, le volontaire sain peut revendiquer une compensation plus élevée, dépassant le simple dédommagement pour constituer une rémunération. La limite entre ces concepts n’est pas évidente et dépend de l’interprétation de chacun en fonction des circonstances ou des contingences. Le défraiement paraît légitime s’il se limite aux dépenses effectuées dans le cadre de la participation à l’essai clinique (déplacement, repas, hébergement, frais en lien direct avec l’astreinte représentée par la participation du volontaire sain à l’essai clinique). La somme allouée, parfois dérisoire pour un promoteur du Nord, peut représenter une véritable aubaine pour le volontaire sain originaire d’un pays à revenu faible ou intermédiaire. À cet égard, la sémantique est d’une grande importance. Si les termes de dédommagement ou d’indemnisation semblent neutres, celui d’incitation que l’on rencontre parfois révèle des intentions plus discutables. Quoi qu’il en soit, la compensation doit rester en rapport avec le niveau socioéconomique du volontaire sain et ne pas constituer une raison de participer à la recherche clinique. En outre, la compensation financière ne doit pas induire un sentiment de honte ou un remords parce que son choix est lié à un besoin conjoncturel résultant de la précarité du volontaire sain, ce que le comité d’évaluation scientifique et, surtout, le comité d’éthique devront s’efforcer de faire appliquer [14,15,25]. La France est le seul pays au monde à avoir inscrit dans la loi un montant maximum d’indemnisation pour un participant à la recherche (6 000 €, somme revalorisée tous les 3 ans en fonction de l’inflation)^6^.

En revanche, la compensation financière pour le temps passé, la perte salariale ou les risques encourus relève davantage de la rémunération, ce que l’éthique occidentale rejette. La revendication d’être propriétaire de son corps peut l’amener à exiger un salaire, d’ailleurs légal dans quelques pays d’Europe ou d’Amérique [19]. Le « droit de disposer de son corps » s’inscrit dans une démarche de contestation de l’autorité et des institutions illustrant l’opposition entre le corps inviolable et la personne qui pourrait, en toute légitimité, décider du contraire [16]. Dans les pays d’Afrique subsaharienne, cette attitude est rarement opposée. Selon certaines traditions, l’être humain ne possède pas son corps qui n’est pas un objet que l’on peut instrumentaliser. Il est « l’expression concrète par laquelle la personne humaine réalise son existence historique ». On ne peut s’en défaire sans abuser la dignité de la personne dont il est la substance [24].

Dans une perspective communautaire, le bénéfice sera donc pensé par rapport à la collectivité et non pour le principal bénéfice de la personne. Il se peut, d’ailleurs, que cette revendication soit soutenue par la communauté. Le bénéfice sera plus facilement compris s’il est matériel et accessible rapidement. Ainsi, la perspective d’une amélioration de l’état de santé du groupe dans un avenir hypothétique – par exemple, la protection conférée par une vaccination et l’extension de celle-ci à l’ensemble de ses membres (Encadré) – sera moins motivante qu’une dotation ou un avantage matériel, visible et immédiat, comme la construction, l’équipement ou la rénovation d’un centre de santé et la formation de son personnel. Peut-être parce que la perspective d’un avantage potentiel est moins attrayante qu’un profit immédiat. Il faut souligner que, le plus souvent, le bénéfice est en relation avec l’intervention.

Les notions de bénéfices et de risques, *a fortiori* le rapport entre les deux, résultent d’une analyse personnelle fortement influencée par l’éducation et la culture. Le médicament répond à une vision du monde et, en particulier, de la maladie. Ces notions sont donc fondées sur les croyances et perceptions culturelles et religieuses de la société. L’efficacité thérapeutique, dont dépend fortement le bénéfice, n’est pas interprétée de façon identique dans les médecines traditionnelle et occidentale. L’efficacité d’un médicament traditionnel est fondée sur des observations indépendantes relevant davantage de la persuasion ou de la foi que de critères objectifs analysés statistiquement selon une méthodologie rigoureuse [29]. L’absence de résultat d’un remède n’est pas rattachée à son inefficacité ou à des raisons pharmacologiques. Au-delà d’une terminologie souvent ambigüe ou mal définie, les entités nosologiques entre médecines traditionnelle et moderne ne coïncident que rarement. Indépendamment du traitement symptomatique éventuellement efficace dans les deux types de médecine, celles-ci se distinguent l’une de l’autre par le traitement étiologique de l’affection qui s’exerce sur des causes de la maladie de nature différente : mystique ou religieuse pour la première, physiopathologique pour la seconde. De même, la notion de risque thérapeutique n’est pas formalisée en médecine traditionnelle pour laquelle les effets indésirables sont rapportés à l’origine de la maladie et non au traitement. En outre, le risque est souvent sous-estimé, voire dénié, par les sujets se pensant en bonne santé. Le sujet familier de la médecine traditionnelle comprend mal les précautions prises par l’investigateur pour justifier l’essai, vérifier – donc suspecter – l’efficacité du produit qu’il promeut et évoquer des risques d’intolérance [5]. Dans une approche communautaire, davantage que dans une société occidentale, elle procède du principe d’autorité : l’efficacité du traitement proposé n’est pas contestée et sa tolérance n’est pas mise en doute [5]. C’est donc sur un autre plan que la motivation du volontaire sain s’exercera entre, d’une part accepter de participer au nom du bénéfice collectif ou de l’avancement de la science et, d’autre part une compensation matérielle. L’estimation du risque et des dommages éventuels, qui constitue un des objectifs essentiels de l’essai clinique (particulièrement au cours de la phase 1), reste difficile à anticiper et relève d’un comité indépendant du tandem promoteur-investigateur et des participants, plus à même d’en fixer les contours et les compensations.

## Consentement informé

Le consentement nécessite au préalable une information sincère, objective, complète, compréhensible et, si besoin, traduite en langues locales [21], ce qui n’est pas propre au volontaire sain ni au patient du Sud. Encore faut-il que le sens soit clair, tant au niveau du vocabulaire que des concepts, notamment nosologiques.

Le contenu de l’information diffère en ce qui concerne la balance bénéfices/risques. Outre qu’elle doit explicitement rappeler le déséquilibre observé chez le volontaire sain, la formulation doit considérer la finalité de l’expérimentation qui est différente entre le patient et le volontaire sain. Selon le contexte, il faudra trouver un compromis entre la participation à la recherche clinique pour le bénéfice exclusif de la collectivité et du tandem promoteur-investigateur [13,18] ou une rétribution. Pour l’éthique occidentale, aucune des deux n’est acceptable. D’un côté, il ne peut y avoir de soumission de l’éthique à la recherche scientifique [2,10]. De l’autre, l’inviolabilité et l’indisponibilité du corps humain font largement consensus, même si certaines communautés cherchent à les relativiser, voire acceptent le principe de la libre disposition de son corps [17].

Si, d’un point de vue communautaire, l’engagement individuel pour le bénéfice du groupe est recevable, cela ne doit pas faire oublier que le consentement est individuel. Il n’est pas possible de recruter un volontaire sain qui n’aurait pas l’assentiment de la communauté, pas plus qu’il ne l’est de le recruter sans son accord à titre personnel. La dialectique du consentement oppose, d’un côté le consentement individuel – au risque de couper la personne de son groupe – et, de l’autre le consentement collectif limitant l’autonomie de la personne [5].

En conséquence, l’information de l’autorité dont dépend l’individu doit être préalable^7^ afin de préparer les deux niveaux d’acceptation. La discussion fera apparaître les contraintes définies par la communauté, indépendamment de celles éventuellement présentées par le participant à l’étude clinique [5]. Outre l’illettrisme, la formalisation de l’information et du consentement soulève plusieurs questions.

La langue est un point d’autant plus important qu’il est fréquent dans les pays d’Afrique subsa-harienne que plusieurs langues soient parlées par les participants d’une même étude. Les concepts et leur perception diffèrent selon la culture, ce qui peut être à l’origine de malentendus : les notions d’essai clinique (expérimentation sur l’homme, cobaye), de placebo (faux médicaments), de tirage au sort (loterie) sont à l’origine de débats et d’incompréhensions [5].

La signature du consentement peut être un autre sujet de discorde. Elle est interprétée comme une défiance de l’investigateur à l’endroit du sujet pour qui le consentement oral (la parole donnée) a une valeur définitive supérieure, *a priori*, à la signature qui relève du domaine judiciaire. La signature a été introduite par le pouvoir colonial dans un contexte juridique; elle apparaît inappropriée, tout comme les empreintes digitales encore parfois demandées par le promoteur, dans une consultation médicale où la relation est fondée sur la confiance, voire la foi, envers le thérapeute. Il ne s’agit pas d’un compromis à l’issue d’une négociation, mais d’une adhésion qui ne nécessite pas de signature.

Il arrive que le sujet ne souhaite pas participer à une étude clinique pourtant acceptée par la communauté qui a préconisé son inclusion. Cette situation particulièrement inconfortable pour le volontaire sain doit être anticipée pour l’aider à trouver une échappatoire. Il faut lui éviter de donner l’impression qu’il s’affranchit d’un devoir social ou culturel ou, simplement, d’une décision collective. Le « refus honorable » consiste à introduire un critère de non-inclusion invérifiable par l’investigateur comme par le groupe, conduisant à une impossibilité de participer à l’étude [12]. Ce peut être, par exemple, un voyage qui doit être accompli pendant l’étude, un désir de grossesse ou le refus d’une contraception empêchant l’inclusion. Cela suppose toutefois que le sujet puisse identifier et utiliser cette ressource au moment de l’expression du consentement et qu’il ne soit pas réfutable par la communauté ou l’autorité. Dans l’exemple du voyage, la non-inclusion doit être décidée si l’absence est prévue pendant l’essai et que le voyage ne peut être, ni reporté, ni délégué à quelqu’un d’autre.

## Justice ou équité du recrutement d’un volontaire sain

Le principe de justice préconise une égale répartition des contraintes et bénéfices entre les participants ou, si l’on préfère, l’obligation de traiter de la même façon les personnes de mêmes conditions. Le principe de justice peut être remplacé par celui d’équité, qui se préoccupe des modalités de répartition du bénéfice conditionnées par l’urgence et les priorités, ainsi que par la faisabilité et l’acceptabilité des mesures, notions plus qualitatives et pragmatiques que quantitatives et égalitaires [32]. Cela s’applique à l’information délivrée aux participants à l’étude, avec les difficultés linguistiques évoquées ci-dessus.

Par ailleurs, il implique de conduire les recherches sur des personnes concernées par celles-ci ou, au moins, sur des sujets représentatifs de celles qui pourront en tirer un avantage. Dans la plupart des études cliniques intéressant les volontaires sains, les principes de justice et d’équité sont peu questionnés puisque leur contribution porte généralement sur des objectifs généraux de recherche clinique et non sur des aspects spécifiques touchant les personnes. Cependant, les volontaires sains sélectionnés pour les phases précoces de développement de nouveaux traitements ou vaccins devraient être plus représentatifs de la diversité de la population qu’ils ne le sont habituellement. Par précaution, certaines catégories de sujets en sont exclues : enfants, senior, femmes en âge de procréer ou membres de groupes minoritaires. Cette réserve opposée systématiquement par l’éthique occidentale n’est pas toujours acceptée par les sujets concernés par l’essai clinique proposé qui revendiquent d’y participer. De plus, elle réduit la validité externe des essais cliniques dont les résultats ne sont pas applicables aux sujets non représentés. Il convient donc de protéger les personnes vulnérables sans toutefois les exclure systématiquement des études cliniques.

L’inclusion des volontaires sains doit résulter d’un consensus entre les responsables de la recherche clinique et la population où elle est menée. Si l’on doit recueillir l’accord de cette dernière, inversement, il ne faut pas qu’elle puisse imposer des personnes contre l’avis de l’investigateur ou du promoteur.

## Rôle des comités d’évaluation scientifique et des comités d’éthique

La participation de volontaires sains dans la recherche clinique doit être examinée avec attention par les divers comités d’évaluation, notamment le comité d’évaluation scientifique et le comité d’éthique. Un comité institutionnel, comme le sont certains comités d’évaluation scientifique et parfois comités d’éthique, peut avoir un conflit d’intérêt, surtout s’il s’agit de la même institution que le promoteur et/ou les investigateurs. En outre, il est indispensable que les membres de ces comités soient informés – voire formés – aux spécificités de la participation du volontaire sain à la recherche clinique. Cet aspect est généralement peu développé dans les lignes directrices éditées à ce jour. La charte VoIREthics y fait spécifiquement référence dans plusieurs de ses articles.

L’article 3 propose de développer la représentation des volontaires sains dans l’ensemble du processus de recherche en favorisant la création d’associations de volontaires sains et leur participation au sein des comités d’évaluation scientifique et d’éthique, notamment dans les pays à faibles revenus et ressources pour éviter le risque d’exploitation des personnes.

L’article 5 se réfère directement à l’expertise, la formation et les ressources des comités d’évaluation scientifique et éthique nécessaires à l’intégrité et la qualité des essais cliniques. Il souligne, d’une part la nécessité d’une compétence appropriée pour évaluer les risques spécifiques encourus par les volontaires sains et, d’autre part la participation au comité de membres d’association de volontaires sains pour leur apporter un éclairage pertinent. L’article 8 concerne la sécurité et le bien-être des volontaires sains face aux éventuelles conditions et contraintes nécessitées par l’étude. Elles doivent être analysées et validées par les comités d’évaluation scientifique et d’éthique afin d’éviter tout risque de préjudices non-physiques.

L’article 13 rappelle que la compensation financière doit être équitable, raisonnable et appropriée. Dans le cadre de la recherche clinique au Sud, cette recommandation revêt une importance particulière. La compensation financière peut conduire à des dérives inacceptables. Elle incite à veiller à la pertinence des bénéfices individuels et collectifs et à leur adéquation avec le protocole de recherche comme, par exemple, les encouragements favorisant la compliance.

L’article 15 insiste sur la surveillance que doivent exercer les comités d’évaluation scientifique et d’éthique lors du déroulement de l’essai clinique puis après la fin de celui-ci, pour permettre un signalement approprié des éventuels problèmes en garantissant qu’ils seront considérés et pris en charge sans représailles.

Ces articles soulignent clairement que les avis scientifiques ou éthiques préalables à la recherche clinique doivent être suivis d’une surveillance pendant l’essai clinique et après la fin de celui-ci pour identifier les problèmes et obliger promoteurs et investigateurs à les résoudre.

## Conclusion

Jusqu’à présent, les volontaires sains n’ont pas bénéficié d’une attention spécifique, distincte de celle concernant les patients inclus dans les études cliniques. La charte VolREthics couvre l’ensemble des aspects liés à la participation des volontaires sains. Elle synthétise un ensemble de recommandations soumises à une large discussion pour être adaptées en fonction des contextes culturels, législatifs et règlementaires. Elle souligne le rôle essentiel des volontaires sains et la nécessité d’optimiser le recours à leurs services tout en limitant les excès. Le déséquilibre du rapport bénéfices/risques, dû à l’absence d’avantage individuel direct et à la difficulté d’évaluer les inconvénients, constitue un risque patent. En particulier, il convient de remédier au danger d’exploitation des volontaires sains vulnérables ou d’écarter ceux qui exigent une rétribution quel qu’en soit le motif.

Au Sud, la perspective communautaire modifie l’autonomie du sujet et la perception du rapport bénéfices/risques qui doivent être anticipées de façon explicite dans l’information préalable délivrée au volontaire sain. L’objectif n’est pas de privilégier une éthique particulière ni de promouvoir des procédures médianes – une sorte de plus petit dénominateur commun –, ce qui serait illusoire et contreproductif, mais de rassurer le sujet et de respecter ses convictions ou contraintes morales. Il s’agit de considérer tous les principes éthiques, d’où qu’ils viennent, pour en faire une synthèse consentie par toutes les parties prenantes de l’étude. À cet égard, il est essentiel de soumettre le protocole de recherche aux comités d’éthique respectifs des pays – lorsqu’ils sont différents – dont dépendent le promoteur, l’investigateur et les participants à la recherche clinique.

## Sources de financement

Cet article n’a pas nécessité de financement.

## Déclaration de liens d’intérêt

Aucun lien d’intérêt n’a été déclaré.

1Il s’agit le plus souvent des phases I des essais cliniques au cours desquelles les volontaires sains sont indemnisés. Cependant, des volontaires sains peuvent participer à des phases ultérieures sans recevoir d’indemnisation.2https://www.inserm.fr/wp-content/uploads/Inserm_ VolREthics_Charte_francais_V2.pdf3Séance du 26 novembre 2024. Voir: https://pod.inserm.fr/video/2250-16-jean-philippe-chippaux-discutantmp4/4En France, la loi Huriet-Serusclat se fonde sur le parfait équilibre du rapport bénéfices/risques, ce qui n’est jamais le cas pour le volontaire sain. Elle a été complétée par l’arrêté du 15 février 2023 (voir note de bas de page 8).5Sauf exception, comme dans le cas d’un essai vaccinal – exclu de la charte VolREthics –, où le sujet bénéficie, si le vaccin est efficace, d’une immunisation contre une maladie.6Arrêté du 15 février 2023 relatif au montant maximal des indemnités en compensation pour contraintes subies qu’une personne peut percevoir au cours d’une même année pour sa participation à une recherche impliquant la personne humaine, un essai clinique, une investigation clinique ou une étude des performances. https://www.legifrance.gouv.fr/jorf/id/JORFTEXT000047233922.7En outre, elle ne peut se substituer à l’avis d’un comité d’éthique, ni à l’autorisation administrative de l’autorité politique.
