# Supplementary material for: Healthy volunteers and clinical research in the South: a community-based approach
Source: Med Trop Sante Int. 2026 Feb 23;6(1):mtsi.v6i1.2026.823. [Article in French] doi: 10.48327/mtsi.v6i1.2026.823 (PMC13216901; doi:10.48327/mtsi.v6i1.2026.823)
Supplement: Related texts

# Research article: Healthy volunteers and clinical research in the South: a community-based approach

## Introduction

The participation of physically and mentally healthy subjects is essential to many areas of health research, especially clinical trials. A healthy volunteer is defined as a participant in clinical research who does not require treatment or medical care, regardless of gender or age. However, these factors may affect the risks and specificities of the investigation. This article does not address the objectives of clinical research or the specific pathologies involved.

Healthy volunteers contribute to scientific progress in the clinical development of health products^1^. They enable the collection of reference information without pathology interfering with the product or biasing the results. During the development of preventive or therapeutic interventions, healthy volunteers measure the safety, adverse effects, and pharmacokinetics of the intervention before it is administered to patients. Subsequent phases will address other characteristics or properties, such as acceptability, dosage, applicability, and therapeutic combinations. These phases will involve non-healthy subjects, with rare exceptions that do not alter the scope of this article. In some cases, such as vaccine trials, healthy volunteers provide the first evidence of efficacy. Based on these preliminary results, further clinical trials may be authorized. Despite their importance in prevention and prophylaxis studies and phase I therapeutic trials, the specific terms of healthy volunteers’ participation are rarely specified. Most national and international recommendations do not distinguish healthy volunteers from patients included in clinical studies [25,27]. However, healthy volunteers differ from patients in that they receive no direct medical benefit and may experience harm to their well-being or health, creating an imbalanced risk-benefit ratio. Additionally, the prospect of financial compensation makes them vulnerable to exploitation [8,16,19].

The VoIREthics charter^2^, proposed by Inserm and its international partners, outlines the conditions for including healthy volunteers in clinical studies. This includes their informed consent after providing detailed information about the study’s objectives, protocol, and expected results. The charter comprises 15 articles that supplement existing provisions on ethics and the protection of healthy volunteers recruited for clinical studies or trials. The charter describes ways to protect volunteers from exploitation, physical or moral harm, and restrictions to their well-being. The charter uses a narrow definition of the term “healthy volunteer.” It focuses specifically on volunteers participating in interventional research involving a health product from which the participants will not directly benefit. This approach is justified because this type of trial may expose healthy volunteers to the greatest risk of harm, exploitation, and endangerment to their health. In this article, however, I will expand the definition of “healthy volunteer” to encompass all subjects participating in clinical interventional research, whether they derive individual benefit or not—including people participating in vaccine trials, who may benefit from them but are excluded from the charter’s definition—since this concept is not universally understood.

The participation of healthy volunteers in clinical research raises questions about four criteria covered by the principles of autonomy, beneficence, and justice: independence from those involved in clinical research, benefits derived from it, risks and harms that must be avoided or compensated for, and prior information.

However, the VoIREthics charter is based on a universalist and individualist approach to ethics, in which the individual takes precedence over the environment and community. Many countries in the Global South have other equally legitimate moral codes that are often very ancient. These codes are not always enshrined in formal texts and sometimes belong to the oral tradition. These codes shed light on certain paradigms of Western ethics, such as personal autonomy and individual benefit. These concepts apply to both patients and healthy volunteers. However, the latter have specific characteristics that are often overlooked in many countries’ laws and regulations, with France being a notable exception since the Huriet-Sérusclat law of 1988.

This article does not aim to oppose, explain, comment on, or justify ethical relativism. Instead, it aims to highlight the perceptions of healthy volunteers, which may differ from those of the sponsor or investigators due to moral and cultural differences.

## Methodological approaches

The comments are taken from a presentation given to the Inserm Ethics Committee during a discussion about the VolREthics charter^3^. They are based on the presenter’s personal experience gained during clinical trials conducted in sub-Saharan Africa (Benin, Cameroon, Guinea, Niger, and Senegal) and South America (Bolivia and Brazil) since the late 1980s. No systematic or targeted bibliographic research was conducted. Instead, references on the philosophical and ethical aspects of clinical research were explored. The comments address all subjects participating in clinical research and highlight the specific characteristics of healthy volunteers. They also reference the influence of certain non-Western moral codes, particularly those of sub-Saharan Africa.

## Autonomy of the healthy volunteer

Provided that the subject has the legal competence, intellectual and physical capacity, and receives appropriate information, the decision to participate in a clinical study is a matter of free will.

However, it would be illusory to think that prior information alone can guarantee a free and informed choice. Complex motivations influence the decision. Some motivations are circumstantial, such as the possibility of remuneration, a topic addressed appropriately in the VoIREthics charter, while others are metaphysical, such as belonging to a community [7]. The latter are not addressed by the VoIREthics charter.

In many societies in the Global South, as well as in some communities in industrialized countries, an individual’s autonomy is constructed in relation to their position and status in the community, particularly the socioeconomic group to which they belong [34]. Whether they are aware of it or not, individuals cannot be separated from the community on which they depend [30]. Based on its interests or convictions, the community makes decisions that limit individual freedom and autonomy in the name of social order [11,31]. The consent required for inclusion in a clinical study is determined by the community to which the individual belongs and sometimes by their place in the ecosystem, though not in absolute terms [27]. Therefore, subjects cannot be totally independent from their groups,, which contrasts with the individual freedom defended by modern ideologies [26]. This perspective alters the perception and application of ethics in Western medicine, where individual decision-making is subordinate to the common good. In any case, this perspective calls into question the universality of ethics and its application, which does not depend on the person, place, or time.

However, this situation is not set in stone. In response to everyday reality, economic and social tensions, political tensions, and globalization, which are disrupting communities and weakening the bonds between their members, particularly in the context of increasing urbanization, multiple sources of information and processes of individualization are developing [4]. Social networks are spreading to all communities in the Global South, perhaps more significantly in urban areas than in rural ones. The circulation of rumors and pressure from conspiracy movements, with their cultural nuances and grievances stemming from ancient or recent history, can significantly impact the decisions of healthy volunteers, even after they are included. Furthermore, misinformation leads to a loss of trust in political and administrative authorities and institutions [20]. The controversy over vaccination (anti-vaxxers), which has intensified during the COVD-19 pandemic, is a clear example of this. It has been developing in sub-Saharan Africa since the start of the Expanded Program on Immunization. Here, too, special attention must be paid to the vulnerability of individuals experiencing an identity crisis.

The principle of autonomy applies to patients and healthy volunteers included in clinical trials. However, healthy volunteers are not motivated by medical reasons. This difference must be considered in the information provided to them and in the conduct of the clinical study. In the absence of direct benefit, healthy volunteers may expect collective benefits for their community, provided this is defined in advance. If the clinical trials validate the new product, these benefits should be organized (Box). Finally, it cannot be ruled out that healthy volunteers may be attracted by material or financial incentives [3].

## Benefit to healthy volunteers and risk assessment

The benefit derived from an experiment in which a subject participates is based on the principle of beneficence. In this regard, Article 8 of the VoIREthics charter emphasizes the well-being of healthy volunteers. Beyond ensuring the absence of physical harm and risk of exploitation, this important notion is relatively novel. Regarding respect for individuals who are sometimes in vulnerable situations, it emphasizes the importance of paying attention to their physical and mental well-being.

Some substitute this principle with the principle of nonmaleficence (“first, do no harm”), which is equally difficult to interpret and apply. Rather than complementarity, we observe an evasion between active engagement (beneficence) and passive avoidance (non-maleficence) [1]. The balance between beneficence and non-maleficence involves assessing the benefit-risk ratio (effectiveness versus adverse effects).

Box: Clinical trial of the hepatitis B vaccine in Niakhar, Senegal

Since the 1960s, the Niakhar population observatory, located in the peanut-growing region of Senegal, has hosted clinical trials [6,9,28]. In the late 1970s, a clinical trial of a new hepatitis B vaccine consisting of a concentrate of AgHBs particles was conducted there to eliminate primary liver cancer, of which it is one of the major causes [22]. Clinical trials of this vaccine (Hevac B Pasteur), developed by Maupas and produced by Institut Pasteur Production, were conducted by the University of Tours and the University of Dakar [22,23]. Although planned at the time the clinical trial was set up, Hevac B Pasteur was not included in the expanded program on immunization due to logistical and financial reasons, which led to tremendous disappointment and severely affected the observatory’s ability to continue its programs [23]. A similar scenario occured during the development of a meningococcal A-C conjugate vaccine in Niamey in the late 1990s. Once again, the inhabitants of Niamey did not have access to the vaccine after its efficacy and good tolerance had been demonstrated in Nigerien newborns.

Societies that promote access to healthcare and medicines through appropriate mechanisms (social security, mutual insurance companies, insurance, etc.) have greater access to treatments validated by clinical trials.

The Declaration of Helsinki grants legitimacy to experimentation if the benefit-to-risk ratio favors the subject^4^. While this balance may favor patients hoping for an improvement in their health or, in some cases, a cure, it is objectively unfavorable for healthy volunteers, who do not expect any direct benefit, but whose health may be impaired by the intervention^5^. This point is not sufficiently emphasized in the latest version of the Declaration of Helsinki [3]. Considering the potential risks and harm that a healthy volunteer may face by participating in a clinical trial, the principle of non-maleficence seems more appropriate. In this regard, insurance companies in sub-Saharan Africa do not cover the risks and constraints of clinical trials, and participants are unaware of their rights in the event of harm. This can lead to difficulties in terms of compensation or redress. Nevertheless, if the community hopes to benefit collectively from the participation of some of its members in clinical research, applying the principle of beneficence to healthy volunteers may satisfy it (Box).

Because they are independent of the study sponsor and investigator, healthy volunteers may claim higher compensation, which constitutes remuneration rather than simple compensation. The distinction between these concepts is unclear and depends on one’s interpretation of the circumstances. Reimbursement seems legitimate if it is limited to expenses incurred in connection with participation in the clinical trial, such as travel, meals, and accommodation costs, as well as costs directly related to the constraints of the healthy volunteer’s participation in the clinical trial. The amount allocated may be insignificant to a sponsor in the North, yet substantial to healthy volunteers in low- or middle-income countries. In this regard, semantics are important. While the terms “compensation” and “indemnification” seem neutral, the term “incentive,” which is sometimes used, reveals more questionable intentions. In any case, the amount of compensation must be appropriate for the socioeconomic status of the healthy volunteer and it should not be a reason they participate in clinical research. Furthermore, the amount of financial compensation should not be so high that it induces feelings of shame or remorse because the choice is linked to a temporary need resulting from the volunteer’s precarious situation. The scientific evaluation committee and, above all, the ethics committee must enforce this [14,15,25]. France is the only country that has enshrined a maximum compensation amount for research participants in law (€6,000, adjusted every three years according to inflation)^6^.

Conversely, financial compensation for time spent, loss of wages, or risks incurred is more akin to remuneration, a concept that Western ethics reject. Claiming ownership of one’s body can lead to demands for wages, which is legal in some European and American countries [19]. The “right to dispose of one’s body” is part of a process of challenging authority and institutions. It illustrates the opposition between the idea of an inviolable body and the idea that a person could quite legitimately decide otherwise [16]. In sub-Saharan African countries, this attitude is rarely opposed. According to certain traditions, human beings do not own their bodies; they are not objects that can be exploited. Rather, they are “the concrete expression through which human beings realize their historical existence.” Disposing of them without consent abuses the person’s dignity [24].

From a community perspective, the benefit will be considered in relation to the community, rather than for the primary benefit of the individual. Moreover, the community may support this claim. The benefit will be more easily understood if it is material and accessible quickly. Thus, the prospect of improving the group’s future health, such as through vaccination – for example, the protection conferred by vaccination and its extension to all members (Box) – will be less motivating than a visible, immediate, material benefit, such as constructing, equipping, or renovating a health center and training its staff. Perhaps this is because the prospect of a potential benefit is less appealing than an immediate gain. It should be noted that, in most cases, the benefit is related to the intervention.

The concepts of benefits and risks, as well as the relationship between the two, result from personal analysis, which is strongly influenced by education and culture. Medicines reflect a worldview, particularly a view of disease. Therefore, these concepts are based on society’s cultural and religious beliefs and perceptions.

Therapeutic efficacy, on which benefits depend, is interpreted differently in traditional and Western medicine. The efficacy of traditional medicine is based on observations that are more a matter of persuasion or faith than objective criteria analyzed statistically according to rigorous methodology [29]. The absence of results from a remedy does not necessarily indicate its ineffectiveness or pharmacological reasons. Beyond the often ambiguous or poorly defined terminology, the nosological entities of traditional and modern medicine rarely coincide. While both types of medicine may be effective in treating symptoms, they differ in etiological treatment, which acts on different causes of disease: mystical or religious for traditional medicine and physiopathological for modern medicine. Similarly, the concept of therapeutic risk is not formalized in traditional medicine, where adverse effects are attributed to the origin of the disease and not the treatment. Furthermore, individuals who consider themselves to be in good health often underestimate or deny risk.

Those familiar with traditional medicine may not fully understand the precautions investigators take to justify trials, verify—and therefore raise questions about—the efficacy of the product they are promoting, and discuss the risks of intolerance [5]. In a community-based approach, more so than in Western society, the principle of authority comes into play: the efficacy of the proposed treatment is not disputed, nor is its tolerance questioned [5]. Therefore, healthy volunteers are motivated by different factors, balancing the collective benefit or advancement of science with material compensation.

One of the key objectives of clinical trials is to assess risk and potential harm, especially in phase 1. Anticipate this remains difficult, so it is the responsibility of an independent committee, that is separate from the sponsor-investigator tandem and the participants. This committee is better positioned to determine the scope and compensation.

## Informed consent

Informed consent requires prior information that is sincere, objective, complete, understandable, and, if necessary, translated into local languages [21], which is not specific to healthy volunteers or patients in the Global South. The information must be clear in terms of both vocabulary and concepts, particularly nosological ones.

The content of the information varies with regard to on the benefit/risk balance. In addition to explicitly mentioning the observed imbalance in healthy volunteers, the wording must consider the purpose of the experiment, which differs between patients and healthy volunteers. Depending on the context, a compromise must be found between participation in clinical research for the benefit of the community and sponsorship, and remuneration [13,18]. Western ethics deems both unacceptable. First, ethics cannot be subordinated to scientific research [2,10]. Second, there is a broad consensus on the inviolability and unavailability of the human body, though some communities seek to relativize this principle or accept the idea of freely disposing of one’s body [17].

Although individual commitment for the benefit of the group is acceptable from a community perspective, this should not obscure the fact that consent is individual. It is impossible to recruit a healthy volunteer without the community’s consent or without their personal consent. The dialectic of consent pits individual consent—which risks cutting the person off from their group—against collective consent, which limits the person’s autonomy [5].

Consequently, the authority to which the individual is subject must be informed in advance^7^ to prepare for both types of acceptance. The discussion will reveal the constraints defined by the community, independent of those presented by the clinical study participant [5]. In addition to illiteracy, formalizing information and consent raises several questions.

Language is an especially important issue because it is common in sub-Saharan African countries for participants in the same study to speak multiple languages. Perceptions of concepts differ according to culture, which can lead to misunderstandings. The concepts of clinical trials (human experimentation and guinea pigs), placebos (fake drugs), and randomization (lottery) are sources of debate and misunderstanding [5].

Signing the consent form may also be a point of contention. It is often seen as a sign of mistrust by the investigator toward the subject. For the subject, verbal consent (a promise) is generally considered more definitive than a signature, which is a legal requirement. Colonial powers introduced signatures in a legal context, but they seem inappropriate in a medical consultation where the relationship is based on trust, or even faith, in the therapist. It is not a compromise reached through negotiation but rather an agreement that does not require a signature.

Sometimes, subjects do not wish to participate in a clinical study that has been accepted by the community that recommended their inclusion. This situation is particularly uncomfortable for healthy volunteers, so it must be anticipated in order to help them find a way out. They must be spared the impression that they are shirking a social or cultural duty or simply a collective decision. An “honorable refusal” involves introducing a non-inclusion criterion that the investigator or group cannot verify, thus preventing participation in the study [12]. Examples include a trip that must be taken during the study, a desire to become pregnant, or a refusal to use contraception. All of these factors would prevent inclusion. However, this assumes that subjects can identify and access this resource at the time of consent, and that community members or authorities cannot refute it. In the case of travel, subjects must be excluded if they will be absent during the trial and cannot postpone or delegate the trip.

## Justice and equity in the recruitment of healthy volunteers

The principle of justice advocates for the equal distribution of burdens and benefits among participants, or the obligation to treat people in similar circumstances equally. However, the principle of justice can be replaced by the principle of equity, which is concerned with distributing benefits based on urgency, priorities, feasibility, and acceptability. These concepts are more qualitative and pragmatic than quantitative and egalitarian [32]. This applies to the information provided to study participants, considering the aforementioned linguistic difficulties.

Furthermore, it implies conducting research on people affected by it, or at least on subjects representative of those who may benefit from it. In most clinical studies involving healthy volunteers, the principles of justice and equity are rarely questioned since their contribution relates to general clinical research objectives rather than specific aspects affecting individuals. However, healthy volunteers selected for the early stages of developing new treatments or vaccines should be more representative of population diversity than they usually are. Certain categories of subjects are excluded as a precaution including children, seniors, women of childbearing age, and members of minority groups. This practice, which Western ethics oppose, is not always accepted by subjects of the proposed clinical trial who demand to participate. Additionally, this reduces the external validity of clinical trials, whose results are not applicable to unrepresented subjects. Therefore, it is necessary to protect vulnerable individuals without excluding them from clinical studies.

The inclusion of healthy volunteers must result from a consensus between those responsible for clinical research and the population in which the research is conducted. While the population’s consent must be obtained, it cannot impose individuals against the investigator’s or sponsor’s opinion.

## Role of scientific review boards and ethics committees

The participation of healthy volunteers in clinical research must be carefully reviewed by various boards, including scientific review boards and ethics committees. An institutional committee, such as a scientific or ethics review committee, may have a conflict of interest, especially if the institution is the same as the sponsor and/or investigators. It is also essential that the members of these committees be informed—and even trained—about the specifics of healthy volunteer participation in clinical research. This aspect is generally underdeveloped in the published guidelines to date. The VoIREthics charter specifically addresses this issue in several articles.

Article 3 aims to enhance the representation of healthy volunteers throughout the research process by encouraging the formation of healthy volunteer associations and their involvement in scientific and ethical review committees, particularly in low-income and low-resource countries, to prevent exploitation.

Article 5 addresses the expertise, training, and resources necessary to ensure the integrity and quality of clinical trials within scientific and ethical review committees. The article emphasizes the need for expertise to assess the specific risks incurred by healthy volunteers and the participation of healthy volunteer association members to provide relevant insight.

Article 8 addresses the safety and well-being of healthy volunteers in the context of possible study conditions and constraints. Scientific and ethical review committees must analyze and validate these conditions to avoid any risk of non-physical harm.

Article 13 reiterates that financial compensation must be fair, reasonable, and appropriate. This is particularly important in the context of clinical research in the Global South. Financial compensation can lead to unacceptable abuses. Therefore, attention should be paid to to ensure that individual and collective benefits are relevant and consistent with the research protocol, such as incentives to promote compliance.

Article 15 emphasizes that scientific and ethics review committees must monitor clinical trials to ensure that any problems are reported appropriately and considered and addressed without reprisals.

These articles clearly emphasize that scientific or ethical opinions prior to clinical research must be followed by monitoring during and after the clinical trial to identify problems and oblige sponsors and investigators to resolve them.

## Conclusion

Until now, healthy volunteers have not received specific attention, distinct from that given to patients included in clinical studies. The VolREthics charter addresses all aspects related to the participation of healthy volunteers. It summarizes a set of recommendations that have been widely discussed to adapt them to different cultural, legislative, and regulatory contexts. The charter highlights the essential role of healthy volunteers and the need to optimize the use of their services while limiting excesses. The imbalance in the risk/benefit ratio, due to the absence of direct individual benefits and the difficulty of assessing the disadvantages, constitutes a clear risk. In particular, the danger of exploiting vulnerable healthy volunteers or excluding those who demand remuneration for any reason must be addressed.

In the South, the community perspective alters both the subject’s autonomy and the perception of the benefit/risk ratio. These alterations must be explicitly anticipated in the information provided to the healthy volunteer in advance. The goal is not to favor a particular ethic or promote median procedures, which would be illusory and counterproductive. Rather, the goal is to reassure subjects and respect their moral convictions or constraints. All ethical principles must be considered, regardless of their origin, to produce a synthesis agreed upon by all stakeholders in the study. In this regard, if they differ, it is essential to submit the research protocol to the ethics committees of the countries to which the sponsor, investigator, and participants belong.

## Funding

This article did not receive any funding.

## Conflicts of interest

Th author declares no conflicts of interest

1This most often occurs in Phase I clinical trials, for which healthy volunteers are compensated. However, healthy volunteers may participate in later phases without receiving payment.2https://www.inserm.fr/wp-content/uploads/Inserm_ VolREthics_Charte_francais_V2.pdf3Session of November 26, 2024. See: https://pod.inserm.fr/video/2250-16-jean-philippe-chippaux-discutantmp4/4In France, the Huriet-Serusclat law is based on a perfect balance between benefits and risks, which is never the case for healthy volunteers. It was supplemented by the decree of February 15, 2023 (see footnote 8).5With some exceptions, such as in the case of a vaccine trial - excluded from the VolREthics charter - where the subject benefits, if the vaccine is effective, from immunization against a disease.6Decree of February 15, 2023 on the maximum amount of compensation for constraints suffered that a person may receive in a single year for participating in research involving human subjects, a clinical trial, a clinical investigation, or a performance study. https://www.legifrance.gouv.fr/jorf/id/ JORFTEXT000047233922.7Furthermore, it cannot replace the clearance from an ethics committee or the administrative authorization of the political authority.
